# Differentiating Delayed Esophageal Clearance From Reflux in Scleroderma

**DOI:** 10.7759/cureus.11553

**Published:** 2020-11-18

**Authors:** Anusha Pasumarthi, Sheena Mago, Promila Banerjee, Micheal Tadros

**Affiliations:** 1 Gastroenterology, Albany Medical Center, Albany, USA; 2 Internal Medicine, University of Connecticut Health Center, Farmington, USA; 3 Gastroenterology and Hepatology, Hines Veterans Administration Hospital, Hines, USA; 4 Gastroenterology and Hepatology, Albany Medical Center, Albany, USA

**Keywords:** scleroderma, gerd, dysphagia, reflux, endoscopy, manometry

## Abstract

Abnormal acid exposure to the esophagus and esophageal dysmotility leading to symptoms of refractory reflux and dysphagia are common findings amongst patients with advanced systemic scleroderma (SSc). Although treatments and diagnostic methods for esophageal disease in the setting of SSc are currently limited to those used for gastroesophageal reflux disease (GERD), certain advancements in diagnostic testing allow potential for improved detection of the exact etiology and clinical management. Through the lens of a case presentation, we found that while GERD is usually diagnosed with high acid exposure from decreased lower esophageal sphincter tone, the high esophageal acidity seen in scleroderma can be attributed to esophageal hypo-motility, leading to fermentation of food residue.

## Introduction

Systemic scleroderma (SSc) is a connective tissue disorder affecting various organ systems including the gastrointestinal, renal, integumentary, pulmonary, and cardiovascular systems. Esophageal dysfunction is present in 50-90% of patients with diffuse scleroderma, which is second only to its manifestations in the skin as the most commonly affected organ [1]. Although patients present with typical reflux symptoms of heartburn and dysphagia, the hypothesized pathophysiological etiology consists of a multitude of factors including vascular, neurological, and musculoskeletal. This combination of functional abnormalities leads to “scleroderma esophagus” (SScE) usually presenting with heartburn, dysphagia, and regurgitation of food, however 30% of patients are asymptomatic [2].

Given the similar symptomatology to gastroesophageal reflux disease (GERD), SScE follows a similar diagnostic approach and treatment plan, including initiating a proton pump inhibitor (PPI) to control acid reflux and minimize damage to the lower esophagus. Although the manometry readings and upper endoscopy findings are similar in SScE to that of GERD, more targeted and tailored management strategy guidelines may benefit these patients.

We present a case of a patient with SScE who exhibited refractory symptoms despite maximum acid-suppressing therapy, where the pH impedance testing aided in determining management.

## Case presentation

**Figure 1:**
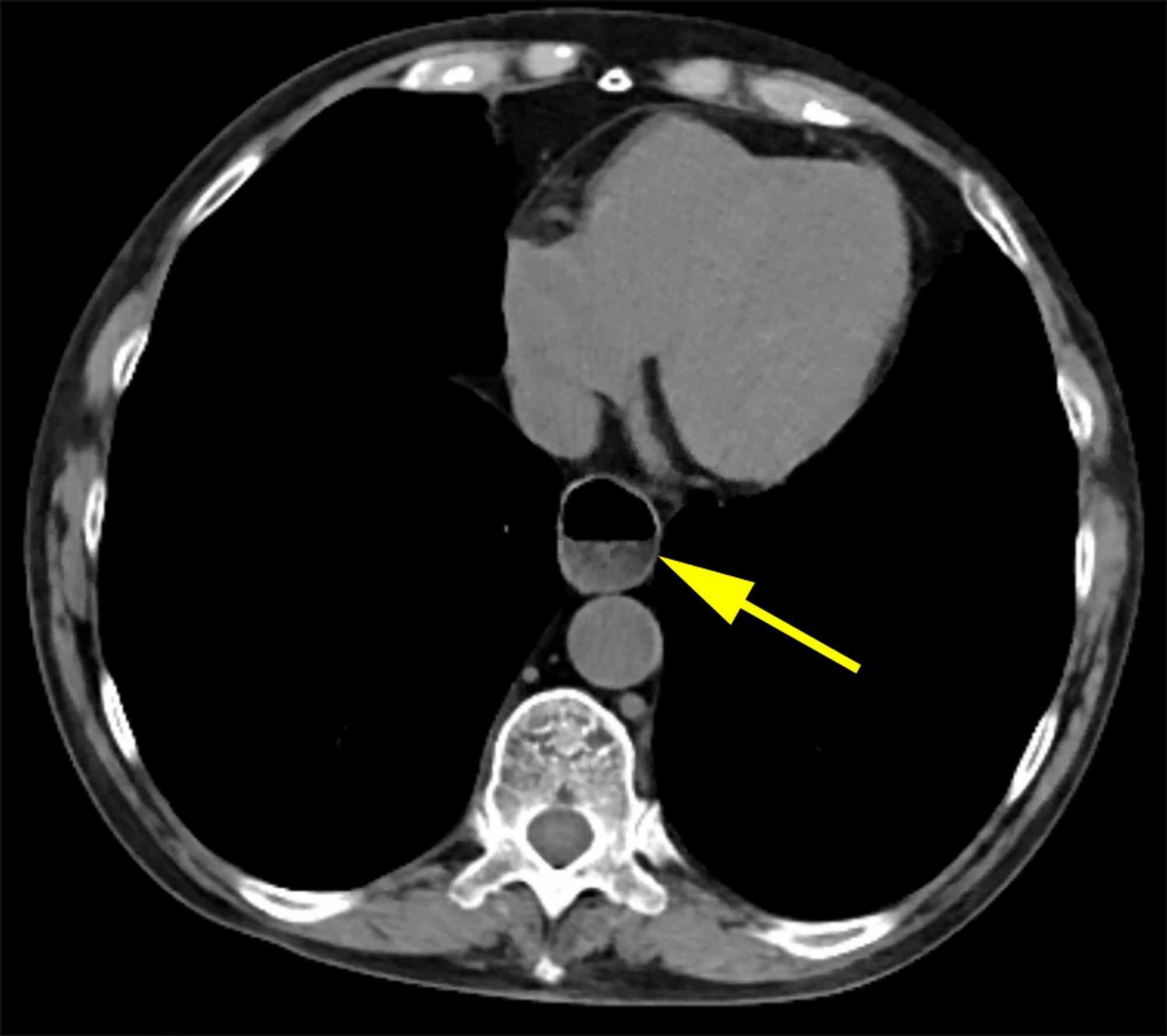
CT scan demonstrating a fluid-filled dilated esophagus

A 70-year-old female with a history of advanced SSc presented with severe retrosternal burning which was at its worst at night and after meals. The patient otherwise denied any history of chest pain, dysphagia, or post-prandial fullness. She was initially treated with 60 mg of dexlansoprazole and famotidine 20 mg twice daily, but continued to have refractory reflux despite maximum acid-suppressing therapy. She underwent a computed tomography (CT) scan of chest and abdomen that revealed a fluid-filled dilated esophagus (Figure 1).

**Figure 2:**
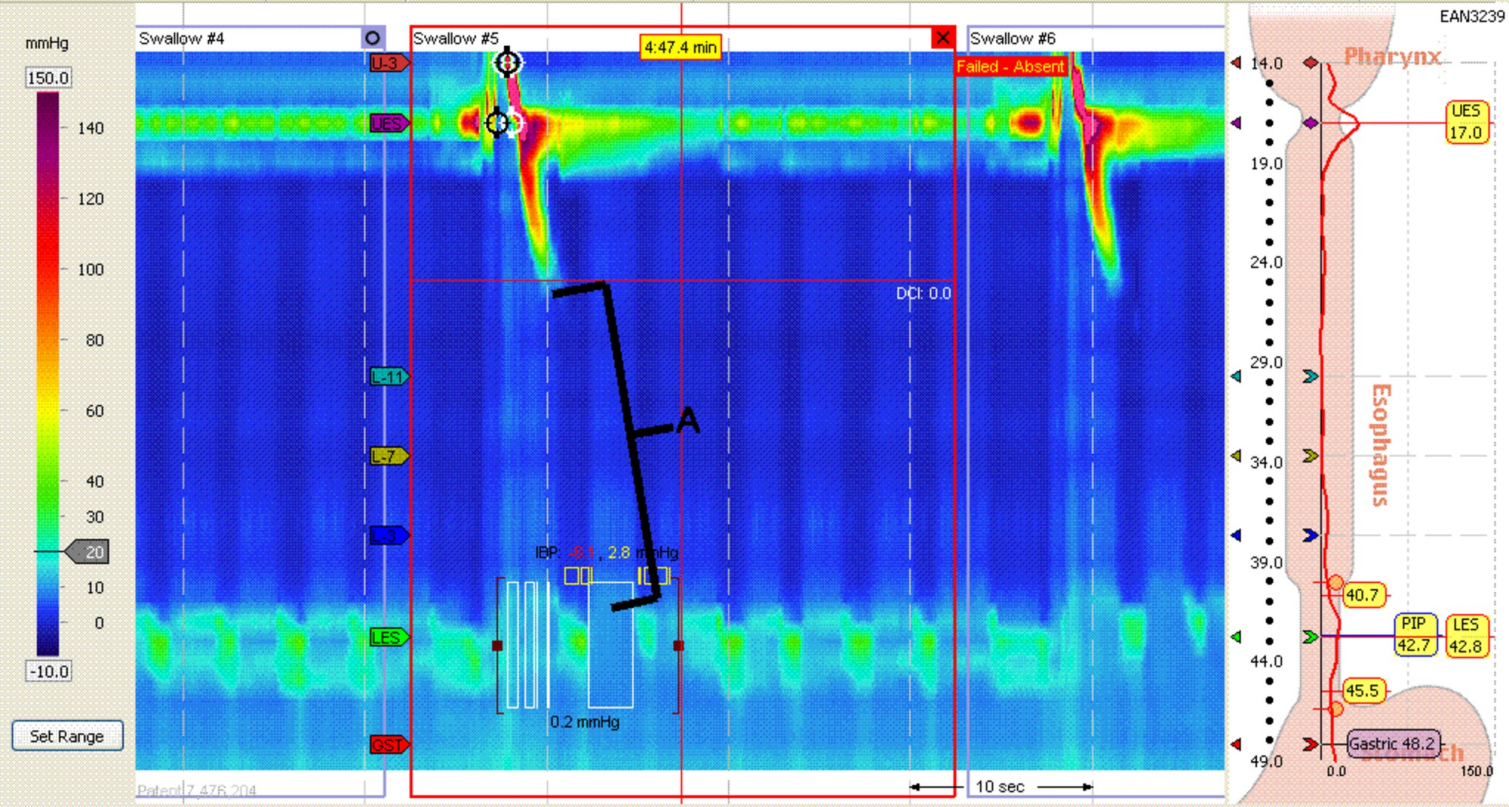
Esophageal high-resolution manometry demonstrating (A) absent esophageal contractility along the length of the esophagus

**Figure 3:**
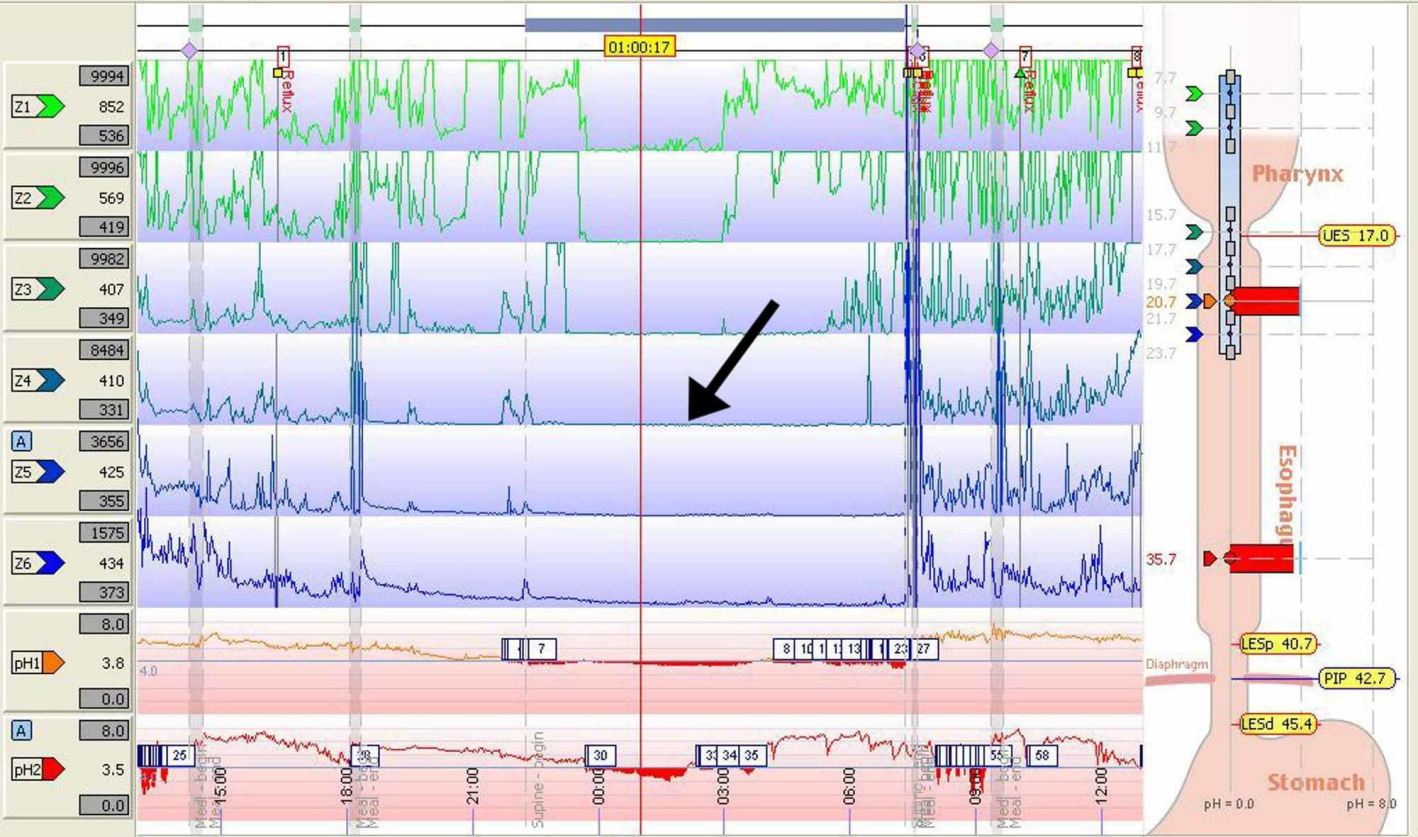
Absent classical saw tooth appearance of esophageal body waves

High-resolution manometry (HRM) showed absent esophageal peristalsis with an integrated relaxation pressure (IRP) of 1.2 mmHg and resting basal pressure of 8.5 mmHg (Figure 2, 3). This was followed up by a 24-hour trans-nasal catheter pH impedance study conducted on dexlansoprazole 60 mg, which confirmed low impedance in the esophagus.

**Figure 4:**
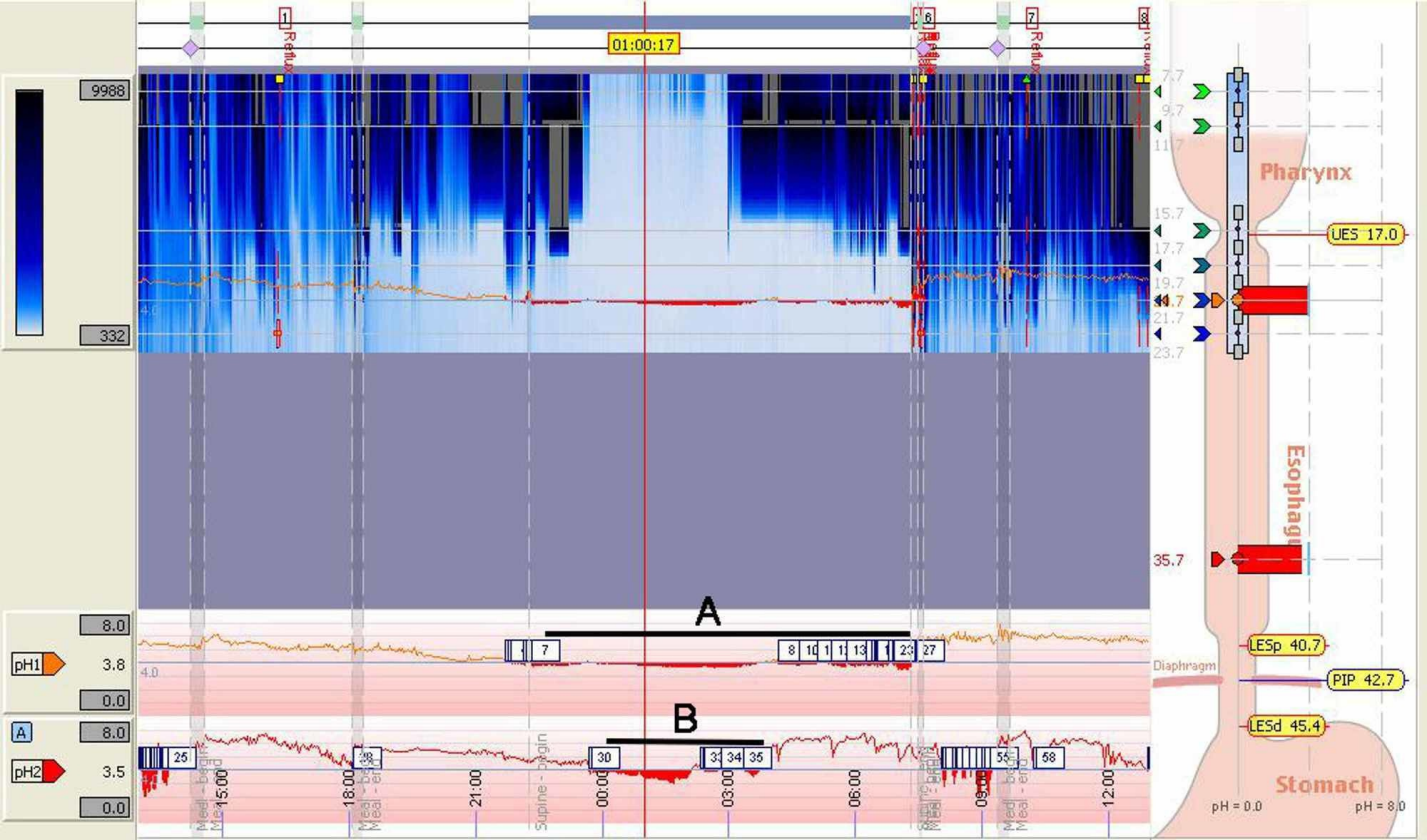
24-hour impedance pH showing low basal impedance and recumbent position acid exposure at the (A) distal esophagus was much more prolonged than (B) nocturnal gastric acidity

Despite having only 36% gastric acid exposure, the patient showed markedly abnormal gastric acid exposure in the distal esophagus with 94% at recumbency and 3% at upright positioning (Figure 4). In the recumbent position, acid exposure at the distal esophagus was more pronounced and prolonged compared to the nocturnal gastric acidity, indicating that esophageal stasis in the setting of supine positioning was likely a significant contributory factor in reflux symptomatology of this patient. The patient also demonstrated abnormal weak acid reflux activity by impedance testing. However, this was difficult to interpret in the setting of generalized low impedance. 

Comprehensive testing with HRM and 24-hour pH impedance demonstrated hypo-motility, prolonged acidic exposure in the distal esophagus compared to the stomach, and increased esophageal acid exposure in the recumbent position. These findings indicated that esophageal stasis in the setting SSc leading to fermentation of food residue was likely the etiology of our patient's reflux symptomatology. She was treated conservatively with lifestyle changes which included avoiding late-night meals, remaining upright after meals, elevation of the head during sleep, using a wedge at night, and taking dexlansoprazole in the evening. On follow-up, her reflux symptoms were controlled and her dose of dexlansoprazole was able to be adjusted to a reduced dose of 30 mg.

## Discussion

Scleroderma involving the gastrointestinal system causes a high prevalence of abnormal esophageal acid exposure and esophageal dysmotility leading to symptoms of refractory reflux and dysphagia. The current therapeutic guidelines of SScE fall closely in-line with GERD, however scleroderma has a more complex and intricate pathophysiology, with diffuse dysregulation of autonomic control leading to hypokinetic peristalsis and decreased esophageal clearance. 

Over 80% of patients with scleroderma have been identified on 24-hour pH monitoring to have abnormal reflux [3]. The impact of PPI therapy on the alteration of pH in scleroderma patients has not been as extensively studied as in GERD patients, although prior randomized control trials of patients with SSc who underwent esophageal manometry with acid clearance tests demonstrated that the acid clearance time was positional [4-5]. It is important to realize that the overall rate of false positives with simple pH monitoring for asymptomatic scleroderma patients with relative bolus stasis becomes another confounding variable in using the overall acid exposure to assess the severity of the disease or acid-related injury to the esophagus [6]. Given that delayed esophageal clearance, due to failed or ineffective esophageal contractions, can lead to a dilated distal esophagus and esophageal stasis-related acidity we can use pH impedance testing to sense food bolus presence which can provide immense insight for scleroderma patients. Nevertheless, if the scleroderma-related neurological degeneration is advanced enough to cause extremely low baseline impedance, the utility of pH-impedance testing may be limited in SSc patients. Thus, for evaluation of baseline electrical and contractile activity of SScE, esophageal manometry should also be performed [6].

In our patient, the HRM (Figure 2) demonstrated findings consistent with scleroderma as there was pressure noted upon oropharyngeal swallowing of the food bolus. However, no peristalsis was noted throughout the entirety of the esophagus. Overall, as seen in Figure 4, there was low impedance and the patient had a prolonged period of esophageal acidity when compared to the stomach. The extended time of acidity in the esophagus versus the stomach lends support to our theory that the patient’s symptoms were likely not from a reflux episode, but from esophageal stasis [7].

Accordingly, delayed esophageal clearance should be considered as the primary role in patient symptomatology when the following are encountered; dilated esophagus with retained secretions noted during esophagogastroduodenoscopy (EGD), dilated esophagus seen on imaging such CT chest or barium study, absent peristalsis and poor bolus clearance on HRM, or a low baseline impedance and prolonged acid exposure in the esophagus when compared to gastric pH on 24-hour pH impedance testing. These findings should ultimately change the treatment approach whereby simple lifestyle and habitual measures such as avoiding meals three hours prior to sleeping, remaining upright after eating, and elevating the head of the bed during sleep can help empty the esophagus and minimize stasis by using the effect of gravity [8]. In our patient, these simple measures helped reduce the dose of PPI and stop other supplemental medications.

## Conclusions

Despite the similarities between the current therapeutic guidelines of SScE and GERD, SSc has a more complex pathophysiology with diffuse dysregulation of autonomic control leading to hypokinetic peristalsis and decreased esophageal clearance. As seen in this case, the hallmark findings of SScE on HRM, EGD, and 24-hour impedance pH monitoring can guide the diagnosis and management of SScE. This case demonstrates that proper utilization of these techniques to determine the underlying etiology of symptoms can assist in successfully tailoring treatment and management techniques for improved symptomatology and quality of life for patients with SSc.
